# Pattern and correlates of cardiac lesions in a group of sub-Saharan African patients on maintenance hemodialysis

**DOI:** 10.11604/pamj.2014.17.3.3422

**Published:** 2014-01-07

**Authors:** Francois Folefack Kaze, Andre-Pascal Kengne, Al Mamy Aboubakar Djalloh, Gloria Ashuntantang, Marie Patrice Halle, Alain Patrick Menanga, Samuel Kingue

**Affiliations:** 1 Department of Internal Medicine and Specialties, Faculty of Medicine and Biomedical Sciences &Yaoundé General Hospital, University of Yaoundé 1, Yaoundé, Cameroon; 2 South African Medical Research Council & University of Cape Town, Cape Town, South Africa; 3 Department of Internal Medicine, Faculty of Medicine and Pharmaceutical Sciences & Douala General Hospital, University of Douala, Douala, Cameroon

**Keywords:** Cardiac lesions, chronic hemodialysis, Cameroon, Sub-Saharan Africa

## Abstract

**Introduction:**

Cardiovascular disease is the leading cause of morbidity and mortality in patients on maintenance hemodialysis. We investigated the pattern and correlates of cardiac lesions in a group of Cameroonians on chronic hemodialysis.

**Methods:**

This was a cross-sectional study conducted at the Yaoundé General Hospital's hemodialysis unit, involving 45 patients (29 men, 64%) on maintenance hemodialysis for at least three months using a native arterio-venous fistula. Cardiovascular risk factors, biological, electrocardiographic and echocardiographic data were collected.

**Results:**

Hypertension (29%), chronic glomerulonephritis (24%) and diabetes mellitus (24%) were the main etiological factors of chronic kidney disease. Blood pressure was controlled in 14 (31%) patients. Nineteen (42%) patients had anemia and 5 (14%) had a calcium-phosphorus product >55 mg^2^/dl^2^. All patients had at least one cardiovascular risk factors with hypertension (95%), anemia (42%) and highcalcium-phosphorus product (42%) being the most frequent. Thirty-eight (84%) patients had at least one cardiac lesion and 11 (29%) had three or more lesions. The cardiac lesions were left ventricular hypertrophy (60%), valvular calcifications (38%), heart failure (36%), conduction disorders (33%), pericardial effusion (13%), valvular diseases (11%) and ischemic heart diseases (2%). Left ventricular hypertrophy was significantly associated with a longer duration on dialysis and low hemoglobin level (both p < 0.047) while cardiac failure and valvular calcifications were associated with advanced age and high interdialytic weight gain (both p <0.05).

**Conclusion:**

Cardiac lesions and cardiovascular risk factors are frequent in these patients receiving sub-optimal dose maintenance hemodialysis despite their younger age, suggesting an increased susceptibility to cardiovascular complications.

## Introduction

Cardiovascular disease (CVD) is the leading cause of morbidity and mortality in patients with chronic kidney disease (CKD) [1–2]. The prevalence increases with the progression of CKD and CVD accounts for up to 50% of mortality among patients receiving long-term dialysis [1–3]. There is a broad spectrum of CVD in CKD patients including alterations in cardiac geometry, atherosclerosis and arteriosclerosis [2].

The left ventricular hypertrophy (LVH) is the most prevalent cardiac lesion in patients with CKD, and is present in over 75% of patients on dialysis [3]. The occurrence of LVH and its evolution to cardiomyopathy, then ultimately cardiac failure, are determined by the high prevalence of traditional and uremia-related cardiovascular risk factors in dialysis patients [3, 4]. During CKD, There is an increased risk of atherosclerosis which is the leading cause of ischemic heart disease in such patients. This is related to the accelerated progression of coronary plaque, greater media thickening and vascular calcification favored by dyslipidemia and mineral bone disease [3–5]. Meanwhile, arteriosclerosis is due to large vessel remodeling and loss of elasticity and compliance, leading to increase pulse pressure and hypertension [3, 4, 6].

In Sub-Saharan Africa (SSA), there is a growing prevalence of CKD which affects predominantly young adults in their economically productive years. These patients are also referred late to nephrologist, prone to acute complications of dialysis, and face infrastructural and financial problems leading to inadequate dialysis in those with end stage renal disease [7–9]. This could contribute to explaining the reported higher prevalence of CVD and cardiovascular risk factors in patients on maintenance hemodialysis in this setting [10–11]. In Cameroon, the provision of only two weekly dialysis sessions of four hours to patients on chronic hemodialysis, added to the above mentioned factors, could favor the occurrence of CVD and cardiovascular risk factors. We therefore conducted this study with the aim of describing the pattern and correlates of cardiac lesions in Cameroonians on chronic hemodialysis.

## Methods

### Settings

This was a cross sectional study of three months duration conducted at the Yaoundé General Hospital (YGH) hemodialysis center from 1^st^ December 2010 to 28^th^ February 2011, corresponding to the period of annual systematic evaluation of chronic hemodialysis patients. At the time this study was conducted, the YGH hemodialysis center was one of the fourth government-funded dialysis centers as described in details previously [9]. These four centers offered hemodialysis, the only method of renal replacement therapy (RRT) available, to the entire country of 20 million inhabitants. The YGH hemodialysis center is equipped with 12 hemodialysis generators that use the Fresenius^®^ 4008S dialysis technology (Fresenius Medical Care, Homburg, Germany), synthetic polysulfone dialysis membrane, and bicarbonate. All patients are dialyzed using standard (unfractionated) heparin with a starting dose of 2500 international units, followed by a maintenance dose of 500 international units per hour during the session. The center operates from Monday to Saturday, from 6:a.m. to midnight (or beyond for emergency cases), and offers to registered patients two hemodialysis sessions of four hours duration each per week. This study was approved by the Cameroon National Ethics Committee, and participants or their next-of-kin provided written informed consent.

### Data collection

We included in the study, all consenting regular (at least eight hemodialysis session per month) chronic hemodialysis patients, aged 18 years and above, who had been on hemodialysis for at least 3 months with a native arterio-venous fistula and performed during annual evaluation at least the electrocardiogram and cardiac ultrasound. Relevant data for the purposes of this study were extracted from dialysis and medical files. Clinical data included age, gender, height, dry weight, underlying renal disease, comorbidities (human immunodeficiency virus infection, viral hepatitis B and C, and Diabetes), past medical history, traditional and uremia-related cardiovascular risk factors, duration in dialysis, ongoing treatment, and average predialysis blood pressure and interdialytic weight gain. The mean of the two later parameters was obtained from the values recorded during the month preceding the inclusion in the study. Biological investigations were done before the second dialysis of the week and included hemoglobin level, total serum calcium, phosphorus, 1-84 parathormone level, uric acid and lipid profile.

The cardiac examination was performed by the same attending consultant cardiologist at the end of the second dialysis session of the week. The cardiac ultrasound was realized using an echocardiograph Hitachi Platform Hi Vision (Hitachi medical corporation, Tokyo, Japan) and standard 12-leads electrocardiogram by Cardi Max Fx-7302 devices (Fukuda Denshi, Tokyo, Japan). The electro-cardiogram was used to diagnose conduction disorders, rhythm abnormalities and ischemic heart disease meanwhile the cardiac ultrasound was used to diagnose heart failure, determine de left ventricular mass and ejection fraction, and evaluate the cavity size, tunics and valves of the heart.

### Definitions and calculations

Anemia was defined by hemoglobin levels of less than 10 g/dl. Dyslipidemia was defined by the presence of either total cholesterol >190 mg/dl, or HDL cholesterol <35 mg/dl, or LDL cholesterol >100 mg/dl. The LVH was defined by a cardiac index of >50g/ m^2,7^ in men or > 47 g/ m^2,7^ in women. It was eccentricor concentric when the relative wall thickness was less or more than 0.42 respectively. All patients with past medical history of diagnosed heart failure treated or not were considered as having heart failure. However, the cardiac ultrasound confirmed the type of heart failure. It was systolic if left ventricular ejection fraction was less than 50% and diastolic depending on the Appleton classification in three stages: abnormalities of relaxation (I), pseudo-normal profile (II) and restrictive profile (III) [12]. Mitral and aortic stenosis was defined by their area less than 2 and 1.8 cm2 respectively. Mitral regurgitation was considered when the four following criteria were mate: seen in two views, jet length above 2 cm in at least one view, velocity above 3 m/s for one complete envelope and pan-systolic jet in at least one envelope. Aortic regurgitation was also considered when the four following criteria were mate: seen in two views, jet length above 1 cm in at least one view, velocity above 3 m/s in early diastole and pan-diastolic jet in at least one envelope.

### Statistical analysis

Statistical analysis used the SPSS^®^ 17 software for Windows^®^ (SPSS Inc., Chicago, USA). We have reported results as mean and standard deviation and count (percentages). Difference between groups was assessed with the use of student t-test and equivalents for quantitative variables and chi-square tests and equivalents for qualitative variables. The level of significance was set at p <0.05.

## Results

### General characteristics of the study population

During the study period, 116 patients were hemodialyzed including 14 (12.1%) patients with acute kidney injury. In the remaining 102 patients on maintenance hemodialysis, 16 (15.7%) had less than 3 months duration in dialysis, 19 (18.7%) patients were dialyzed by catheter and 22 (21.6%) patients did not have electrocardiogram and cardiac ultrasound done during annual evaluation.

The general characteristics of 45 remaining patients (29 men, 64%) who fulfilled the inclusion criteria are presented in Table 1. The main etiological factors for underlying renal disease were hypertension (29%), chronic glomerulonephritis (24%) and diabetes mellitus (24%). Only 14 (31%) patients had controlled blood pressure levels based on values of less than 140/90 mmHg. Two (4%) patients were obese with body mass index above 30 kg/m2. Anemia was present in 19 patients (42%) and 12 (27%) patients were receiving erythropoiesis stimulating agents. In 5 (14%) patients, there was an increased calcium-phosphorus product >55 mg2/dl2 and 19 (42%) patients were treated by phosphates binders. Parathormone levels was measured in 8 (18%) patients with a median value of 718.5 ng/ml, (ranging from 351-1491 ng/ml).

**Table 1:** General characteristics of study population

Parameters	Total
**N (%)**	45 (100)
Mean age (±SD), years	52.7±11.3
Mean duration on dialysis (±SD), months	36.5±23.2
Past history of cardiac lesions, n (%)	19 (42.2)
ACEI/ARA2 treatment, n (%)	24 (53.3)
Mean SBP (±SD), mmHg	149 ± 21
Mean DBP (±SD), mmHg	77 ± 14
Mean dry weight (±SD), kg	66.5±13.2
Mean BMI (±SD), kg/m^2^	22.9±3.4
Mean interdialytic weight gain (±SD), kg	3.1 ± 0.8
Mean hemoglobin level (±SD), g/dl	10.2±1.4
Mean total serum calcium (±SD), mg/dl	8.7 ± 0.9
Mean serum phosphorus (±SD), mg/dl	4.6 ± 1.8
Mean calcium-phosphate product (±SD), mg^2^/dl^2^	39.4 ± 15.4

ACEI, Angiotensin Converting Enzyme Inhibitor; ARA, Angiotensin Receptor Antagonist; BMI, Body Mass Index; DBP, Diastolic Blood Pressure; SBP, Systolic Blood Pressure; SD, Standard Deviation

### Prevalence of cardiac lesions and cardiovascular risk factors

**Figure 1:**
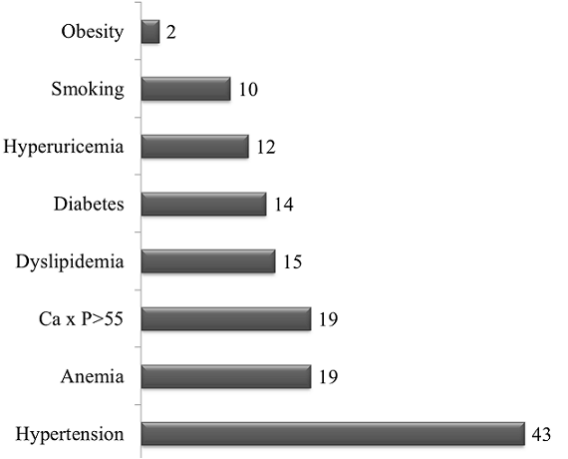
Distribution of cardiovascular risk factors Horizontal bars are the absolute number of patients with each of the cardiovascular risk factors

**Figure 2:**
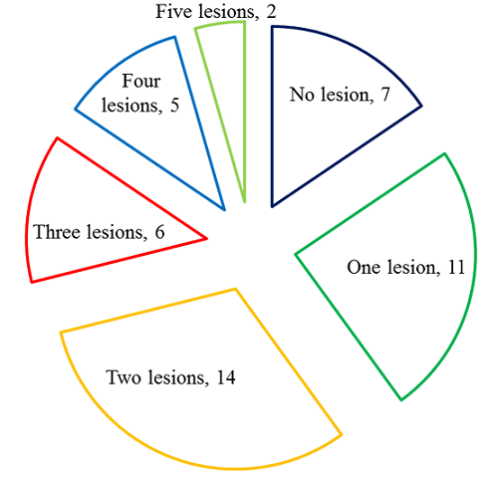
Distribution of cardiac lesions

**Figure 3:**
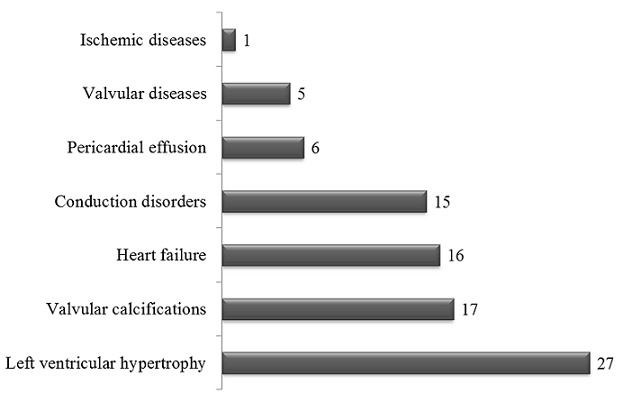
Pattern of cardiac lesions Horizontal bars are the absolute number of patients with each of the cardiac lesions

All the included patients had at least one traditional or uremia-related cardiovascular risk factor (Figure 1). Hypertension (95%), anemia (42%) and increased calcium-phosphorus product (42%) were the most prevalent cardiovascular risk factors. This study revealed that 38 (84%) patients had at least one cardiac lesion with 11 (29%) patients presenting at least three cardiac lesions (Figure 2). As presented in Figure 3, the LVH (60%) was the most prevalent cardiac lesions with 55% being of eccentrictype. The valvular calcifications were seeing on the mitral (47%), the aortic (29%) and both mitral/aortic (23%) valves. We found no case of coronary calcifications. The valvular diseases were mitral regurgitation (50%), aortic stenosis (40%) and tricuspid incompetence (20%). The heart failure was diastolic in 14 (87%) patients of whom 7(50%) had relaxation abnormalities, 6(43%) had pseudo-normal profile and 1 (7%) had restrictive profile. The conduction disorders include 10 (67%) bundle branch block and 5 (33%) atrioventricular block.

### Correlates of cardiac lesions

As presented in Table 2, the presence of LVH was significantly associated with the absence of diabetes, a longer duration on dialysis and low hemoglobin level (all p < 0.047) while the advanced age was associated with cardiac failure and valvular calcifications (all p < 0.039). The results for the 8 patients with parathormone measurements showed an association between the presence of valvular calcifications and the increase level of parathormone (p = 0.031) (data not shown) despite the lack of association with calcium-phosphorus product (p = 0.688) (Table 2).

**Table 2:** Correlates of cardiac lesions, left ventricular hypertrophy, cardiac failure and valvular calcifications

Parameters	Cardiaclesions			Leftventricularhypertrophy			Cardiacfailure			Valvular calcifications		
	No	Yes	p	No	Yes	p	No	Yes	p	No	Yes	p
**N**	8 (17.8)	37 (82.2)		18 (40)	27 (60)		29 (64.4)	16 (35.6)		28 (62.2)	17 (37.8)	
Male sex, n (%)	4 (50)	25 (67.6)	0.427	12 (66.7)	17 (62.9)	>0.999	20 (68.9)	9 (56.3)	0.518	17 (60.7)	12 (70.6)	0.541
Age, years (SD)	48.0 (10.0)	53.7 (11.5)	0.199	54.4 10.2)	51.6 (12.1)	0.425	50.1 (10.0)	57.4 (12.5)	0.039	48.4 (9.7)	59.9 10.4)	0.001
Diabetemellitus, n (%)	2 (25)	12 /32.5)	>0.999	9 (50)	5 (18.5)	0.047	9 (31)	5 (31.2)	>0.999	6 (21.4)	8 (47)	0.101
Smoking, n (%)	2 (25)	8 (21)	>0.999	4 (22.2)	6 (22.2)	>0.999	8 (27.6)	2 (12.5)	0.292	6 (21.4)	4 (23.5)	>0.999
Dyslipidemia, n (%)	2 (25)	13 (31.1)	0.699	8 (44.4)	7 (25.9)	0.218	10 (34.5)	5 (31.2)	>0.999	9 (31.1)	6 (35.3)	>0.999
Duration on dialysis [Q1-Q3]	24 [14-42]	30 [17-54]	0.389	18 [10-38]	41 [26-32]	0.009	30 [16-45]	34 [17-63]	0.602	31 [16-60]	29 [10-44]	0.725
History of cardiac lesions, n (%)	6 (75)	17 (45.9)	0.435	7 (38.9)	12 (44.4)	0.766	10 (34.5)	9 (56.2)	0.212	11 (39.3)	8 (47)	0.757
HIV, HBV or HCV, n (%)	3 (37.5)	9 (24.3)	0.661	3 (16.7)	9 (33.3)	0.308	7 (24.1)	5 (31.2)	0.528	6 (21.4)	6 (35.3)	0.325
IEC/ARA2 treatment, n (%)	3 (37.5)	21 (56.7)	0.443	8 (44.4)	16 (59.2)	0.374	16 (55.2)	8 (50)	0.765	16 (57.1)	8 (47)	0.552
SBP, mmHg (SD)	137 (17)	151 (19)	0.094	145 (22)	151 (21)	0.331	148 (22)	149 (21)	0.908	146 (22)	152 (19)	0.384
DBP, mmHg (SD)	75 (19)	77 (13)	0.609	74 (14)	79 (14)	0.299	79 (14)	74 (14)	0.267	79 (14)	74 (13)	0.274
BMI, kg/m^2^ (SD)	24.0 (2.9)	22.6 (3.5)	0.293	23.0 (2.7)	22.8 (3.8)	0.916	23.6 (3.6)	21.5 (2.7)	0.044	23.4 (3.1)	22.0 (3.8)	0.199
Interdialytic weight gain, kg (SD)	3.4 (0.5)	3.1 (0.9)	0.281	3.1 (0.5)	3.1 (1.0)	0.976	3.3 (0.8)	2.8 (0.8)	0.059	3.3 (0.9)	2.8 (0.7)	0.047
Hemoglobin level, g/dl (SD)	10.4 (1.2)	9.8 (1.4)	0.338	10.5 (1.4)	9.5 (1.3)	0.022	10.1 (1.4)	9.6 (1.5)	0.311	9.8 (1.4)	10.1 (1.3)	0.484
Total serum calcium, mg/dl	8.5 (0.4)	8.7 (1.0)	0.733	8.8 (1.0)	8.6 (0.9)	0.506	8.7 (0.8)	8.7 (1.2)	0.925	8.5 (0.8)	8.9 (1.2)	0.152
Serumphosphorus, mg/dl	5.1 (1.7)	4.5 (1.9)	0.449	4.9 (1.8)	4.3 (1.8)	0.314	4.9 (2.0)	4.1 (1.4)	0.259	4.8 (1.9)	4.3 (1.7)	0.402
Calcium-phosphate product, mg^2^/dl^2^	43.9 (15.0)	38.5 (15.6)	0.450	43.4 (16.1)	36.3 (14.5)	0.172	41.9 (17.1)	35.0 (11.2)	0.200	40.3 (16.0)	38.2 (15.2)	0.688

ACEI, Angiotensin Converting Enzyme Inhibitor; ARA, Angiotensin Receptor Antagonist; BMI, Body Mass Index; DBP, Diastolic Blood Pressure; HIV, Human Immunodeficiency Virus; HBV, Hepatitis B Virus; HCV, Hepatitis C Virus; Q1-Q3, 1^st^-3^rd^ quartiles; SBP, Systolic Blood Pressure; SD, Standard Deviation

## Discussion

This study conducted in young end stage renal disease patients receiving a lower hemodialysis dose than the standard of care revealed a high prevalence of cardiac lesions led by LVH, valvular calcifications and heart failure. These cardiac lesions were associated with advanced age, longer duration on dialysis, higher interdialytic weight gain, anemia and higher parathormone levels, which are similar to patterns reported elsewhere in older patients [3–6, 10, 11]. However, we did not observe coronary calcifications which could be related to the younger ager of patients and shorter duration in dialysis. As reported in the literature, we observed a high prevalence of traditional (hypertension and dyslipidemia) and uremia-related (anemia, increased calcium-phosphorus product) cardiovascular risk factors [3–6, 10–11].

The alteration of cardiac geometry was the most prevalent cardiac lesion mainly in the form of eccentric LVH which was associated with a longer duration on dialysis and anemia as reported in the literature [3, 11, 13]. The increased prevalence of LVH and heart failure could be related to the higher prevalence of hypertension in this study sample which is the leading etiological factor of underlying renal disease. Furthermore, hypertension is one of the main clinical manifestations in patients with glomerulonephritis and diabetic nephropathy. In this setting without a third-payer system implying that drugs and non-subsidized dialysis costs are covered from out-of pocket payments, the association of low hemodialysis dose, long interdialytic intervals with consequent increase in interdialytic weight gain and extracellular volume overload, could explain the high prevalence of uncontrolled hypertension, and hence the development of eccentric LVH, diastolic dysfunction and ultimately cardiac failure [14]. The high prevalence of anemia due to the low use of erythropoiesis stimulating agents in these patient population using arterio-venous fistulae for dialysis constitutes another risk factor for LVH. Anemia is associated with high cardiac output, high stroke volume, increase heart rate and worsening of left ventricular dilation [3, 13, 15–16].

Cardiovascular calcifications may involve the arterial media, atherosclerotic plaques, heart valves and the myocardium in patients with CKD. We found calcifications of the mitral and aortic valves to be associated with advanced age and raised parathormone levels as reported elsewhere [11, 17]. However, unlike many studies, we found no association between valvular calcifications and the calcium-phosphorus product [18]. The reported prevalence of valvular diseases and pericardial effusion was similar to findings by Kane et al in Dakar [11]. These results could be explained by chronic volume overload from under dialysis leading to functional valvular incompetence and pericardial effusion. It has been shown that the progression of calcific aortic stenosis is three times faster in dialysis patients than in general population, thus the recommendation of annual Doppler echo cardio-graphic studies as part of their follow-up [19, 20]. There were few cases of ischemic heart diseases, in line with the results of Kane et al [11]. This could be explained by the younger age of patients, the shorter duration in dialysis and the low frequency of ischemic heart disease in Africans who are prone to LVH and cardiac failure [11].

This study has some limitations including the sub-optimal dose of dialysis delivered to patients which increases their risk for cardiovascular disease. We were unable to perform the effort test which could give an accurate prevalence of ischemic heart disease. The cost of parathormone dosage limited the measurements in all the patients, and hampered our ability of evaluate mineral bone disease in these patients.

## Conclusion

This study has revealed a high prevalence of cardiac lesions and cardiovascular risk factors in this group of young patients receiving sub-optimal dose maintenance hemodialysis. These results suggest the need for cohort studies to assess the impact of hemodialysis on cardiovascular outcomes, and assist the implementation of cardiovascular prevention strategies in this setting.
